# Effect of tanshinone IIA for myocardial ischemia/reperfusion injury in animal model: preclinical evidence and possible mechanisms

**DOI:** 10.3389/fphar.2023.1165212

**Published:** 2023-05-16

**Authors:** Peng-Chong Zhu, Jiayi Shen, Ren-Yi Qian, Jian Xu, Chong Liu, Wu-Ming Hu, Ying Zhang, Ling-Chun Lv

**Affiliations:** ^1^ Department of Cardiology, Lishui Hospital of Zhejiang University, The Fifth Affiliated Hospital of Wenzhou Medical University, Lishui Municipal Central Hospital, Lishui, China; ^2^ Department of Gynaecology and Obstetrics, The First Affiliated Hospital of Jiamusi University, Jiamusi, China

**Keywords:** tanshinone IIA, myocardial ischemia/reperfusion injury, preclinical evidence, possible mechanisms, meta-analysis

## Abstract

**Introduction:** Tanshinone IIA (Tan IIA), the major active lipophilic ingredient of *Radix Salviae Miltiorrhizae*, exerts various therapeutic effects on the cardiovascular system. We aimed to identify the preclinical evidence and possible mechanisms of Tan IIA as a cardioprotective agent in the treatment of myocardial ischemia/reperfusion injury.

**Methods:** The study quality scores of twenty-eight eligible studies and data analyses were separately assessed using the CAMARADES 10-item checklist and Rev-Man 5.3 software.

**Results:** The study quality score ranged from 3/10 to 7/10 points. The present study provided preliminary preclinical evidence that Tan IIA could significantly decrease the myocardial infarct size, cardiac enzyme activity and troponin levels compared with those in the control group (*p < 0.05*).

**Discussion:** Tan IIA alleviated myocardial I/R injury via antioxidant, anti-inflammatory, anti-apoptosis mechanisms and improved circulation and energy metabolism. Thus, Tan IIA is a promising cardioprotective agent for the treatment of myocardial ischemia/reperfusion injury and should be further investigated in clinical trials.

## 1 Introduction

Acute myocardial infarction (MI) is serious consequence of coronary artery disease and the leading cause of death and disability worldwide (Ibáñez et al., 2015; Tsao et al., 2022). The most effective and well-established therapeutic strategy for treating acute MI patients is timely reperfusion by primary percutaneous coronary intervention or thrombolysis, which limits myocardial infarct size, preserves left ventricular systolic function and prevents the onset of heart failure (Bulluck et al., 2016). However, the process of restoring coronary blood flow to the ischemic myocardium can cause myocardial ischemia/reperfusion (I/R) injury, including reperfusion arrhythmias, the no-reflow phenomenon and myocardial stunning (Yellon and Hausenloy, 2007). Several mechanical and pharmacological therapies have been investigated to attenuate I/R injury over the past 30 years (Hausenloy and Yellon, 2013; Thind et al., 2015). Ischemic preconditioning and ischemic postconditioning are two major forms of mechanical strategies that apply transient episodes of myocardial ischemia and reperfusion either before or after the ischemic event, respectively, to protect the heart from I/R injury. (Hausenloy and Yellon, 2007). However, ischemic preconditioning (Heusch, 2013) is not feasible in the clinical setting because of the unpredictability of MI in patients, while the efficacy of ischemic postconditioning (Binder et al., 2015) is still inconclusive in clinical trials. In addition, pharmacological strategies failed to improve clinical trial outcomes (Frank et al., 2012; Perricone and Vander Heide, 2014; Ibáñez et al., 2015). Thus, a novel cardioprotective strategy is needed.

**FIGURE 1:**
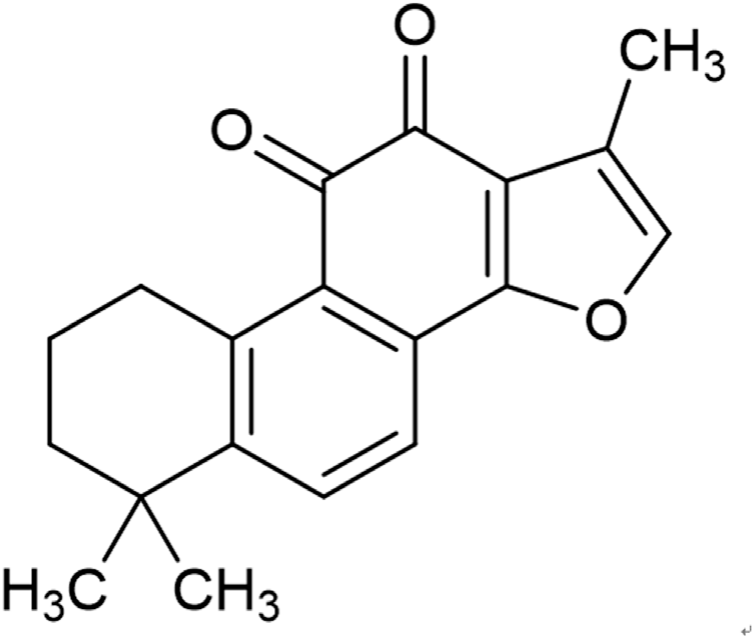
Chemical structures of Tan IIA (C_19_H_18_O_3_, MW 294.34).

*Radix Salviae Miltiorrhizae*, the dried root and rhizome of *Salvia miltiorrhiza Bge.*, a popular Chinese herbal medicine, has the function of activating blood flow circulation and dissipating blood stasis (Guo et al., 2020). It has been widely used in the treatment of various cardiovascular diseases for hundreds of years (Ansari et al., 2021). Tanshinone IIA (Tan IIA) (Figure 1), the major active lipophilic ingredient of *Radix Salviae Miltiorrhizae*, possesses antioxidant, anti-inflammatory (Kai et al., 2011), anti-apoptosis and anti-proliferative pharmacological properties (Shao et al., 2018; Luo et al., 2019). Systematic research reviews of animal studies provide important insights into the validity of animal studies, the precision of the estimated effects and the explanations of underlying mechanisms, and these reviews assist in the determination of whether a certain drug should be evaluated in human clinical trials (Roberts et al., 2002; Sandercock and Roberts, 2002; Briel et al., 2013). Therefore, we reviewed the available preclinical evidence and possible mechanisms of Tan IIA as a cardioprotective agent for myocardial I/R injury.

## 2 Methods

This review complied with the guidelines of the Preferred Reporting Items for Systematic Review and Meta-Analyses (PRISMA) statement (Stewart et al., 2015).

### 2.1 Data sources and search strategy

We searched the following seven databases: PubMed, Cochrane Library, Embase, Wanfang database, China National Knowledge Infrastructure (CNKI), VIP database (VIP), and China Biology Medicine disc (CBM). The search time was limited from inception to the end of September 2022. The search terms used were “danshentong IIA OR tanshinone IIA OR tanshinone” AND “myocardial damage OR myocardial injury OR myocardial infarction OR myocardial ischemia OR myocardial ischemia reperfusion.” The reference lists of potential articles were hand-searched.

### 2.2 Eligibility criteria

To prevent bias, the inclusion criteria were as follows: 1) animal studies *in vivo*; 2) myocardial I/R model induced by coronary artery occlusion; 3) analyzed intervention received Tan IIA treatment only, comparator intervention received vehicle or no treatment; 4) primary outcomes were myocardial infarction (MI) size, cardiac enzymes or cardiac troponin T/I (cTnT/I) level, while secondary outcomes were serum indices or protein levels related to cardioprotection mechanisms. Studies in which other traditional Chinese medicines were administered with other additional pharmacological treatments, studies with no control group, duplicate publications and *in vitro* or *ex vivo* studies were excluded.

### 2.3 Data extraction

Data were extracted independently by two authors, and discrepancies were discussed in group consultation. The following details were recorded: the authors; study time; animal information (species, sex, number, and weight); and interventions, modeling methods, the dose of drugs, and outcomes. When various doses of the drug were used or outcomes were measured at different time points in trials, we adopted the highest dose and the final time measurements.

### 2.4 Risk of bias in individual studies

Quality evaluation of the included studies was conducted by the modified CAMARADES 10-point scoring scale (Macleod et al., 2004; Yu et al., 2017). The modifications are listed as follows: D, blinded induction of model; F, use of anesthetic without significant intrinsic cardioprotective activity; G, appropriate animal model (aged, diabetic, or hypertensive); disagreements were resolved through adjudication by the corresponding author.

### 2.5 Statistical analysis

Meta-analysis was performed using RevMan V.5.3 software. All data abstracted were classified into continuous variables and given an estimate of the combined overall effect sizes by utilizing the standard mean difference (SMD) or mean difference (MD) with the effects model. Heterogeneity and the choice of effects model were assessed using the Cochrane Q statistic test and the I^2^-statistic test. A fixed-effects model was selected when statistical heterogeneity was identified (heterogeneity test, if *I* ^*2*^ *< 50%* and *p < 0.10*), otherwise, a random effects model was used.

## 3 Results

**FIGURE 2:**
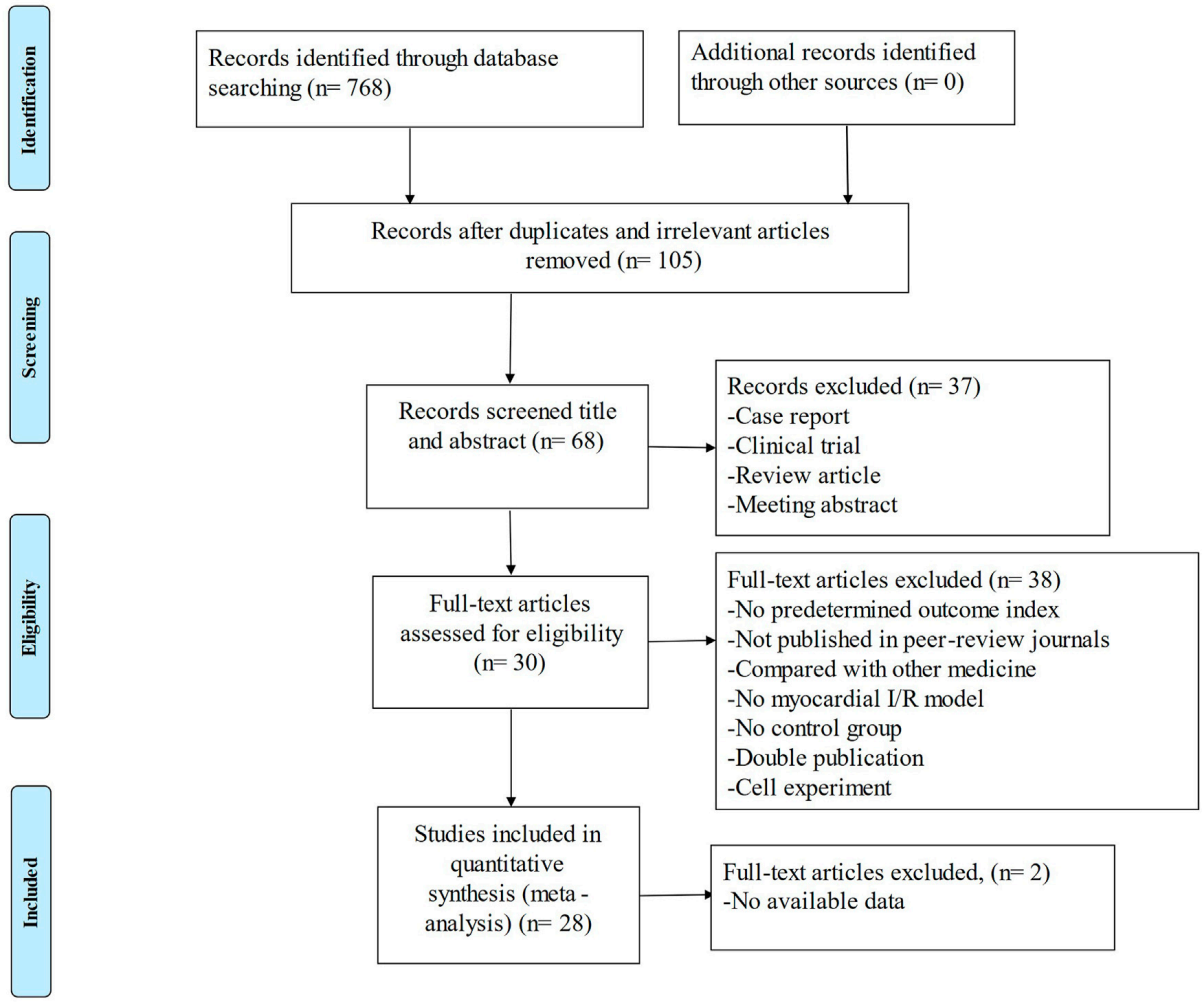
Summary of the process for identifying candidate studies.

After systematically searching the databases, 768 published articles were identified. According to the eligibility criteria, we excluded inappropriate studies, and 28 studies were ultimately included in the systematic review (Figure 2).

### 3.1 Characteristics of included studies

Twenty eight studies (19 studies in Chinese and 9 in English) were published between 1996 and 2021. Sprague‒Dawley (SD) rats, Wistar rats, male/female rabbits, male New-Zealand rabbits, and male/female Japan-Sino hybridization white rabbits were used in these studies. Modeling in these studies included ligation of the left anterior descending coronary artery (LAD), the left ventricular coronary artery (LVA), the left circumflex coronary artery (LCA) and the left marginal branch (LMB). The dosage of TanIIA varied between 3 mg•kg^−1^ and 70 mg•kg^−1^. Evaluated metrics included myocardial infarct size, myocardial cell apoptosis, arrhythmia score, lactate dehydrogenase (LDH), creatine kinase (CK), creatine kinase-MB (CK-MB), cTnI, superoxide dismutase (SOD), malondialdehyde (MDA), glutathione (GSH), glutathione peroxidase (GSH-Px), caspase-3, B-cell lymphoma-2 (Bcl-2), Bcl-2-Associated X (Bax), interleukin-6 (IL-6), tumor necrosis factor-α (TNF-α), aspartate transaminase (AST), high mobility group box-1 protein (HMGB1), amplitude of T wave, ST-segment elevation, ejection fraction (EF), and fractional shorting (FS). The detailed characteristics of the studies are shown in Table 1.

**TABLE 1:** Characteristics of the 28 included studies.

Study (years)	Species (sex, *n* = experimental/control group)	Weight	Model (method)	Anesthetic	Treatment group (method to Tan IIA)	Control group	Outcome index (time)	Intergroup differences
Ma et al., 2017	SD rats (male, 16/16)	180–220 g	Ligation of LAD for 30 min then reperfusion for 180 min	Phenobarbital Sodium (3%)	Gavaged with Tan IIA (20 mg/kg*d), once a day, for 5 days, before establishing model	Gavaged with isasteric normal saline, once a day, for 5 days, before establishing model	1. Infarct size (AAI/AAR)	1. *p* < 0.05
							2. cTnI	2. *p* < 0.05
							3. Cytc	3. *p* < 0.05
Dai et al., 2013	SD rats (male, 8/8)	240–320 g	Ligation of LAD for 40 min then reperfusion for 120 min	20% urethane (5 mL/kg)	Intravenous injected with Tan IIA (30 mg/kg), 3 min before reperfusion and 2 min after reperfusion	Intravenous injected with nothing, 3 min before reperfusion and 2 min after reperfusion	1. Apoptosis index	1. *p* < 0.01
							2. CK	2. *p* < 0.01
							3. LDH	3. *p* < 0.01
							4. SOD	4. *p* < 0.05
							5. MDA	5. *p* < 0.05
Tang et al., 2017	SD rats (male, 10/10)	260–280 g	Ligation of LAD for 30 min then reperfusion for 480 min	3% pentobarbital sodium (30 mg/kg)	Intraperitoneal injected with Tan IIA (30 mg/kg), once a day, for 7 days, before establishing model	Intraperitoneal injected with isasteric normal saline, once a day, for 7 days, before establishing model	1. SOD	1. *p* < 0.01
							2. MDA	2. *p* < 0.01
							3. CAT	3. *p* < 0.01
							4. GSH-Px	4. *p* < 0.01
							5. Nrf-2	5. *p* < 0.01
							6. HO-1	6. *p* < 0.01
Zheng et al., 2010	SD rats (male, 10/10)	200–300 g	Ligation of LAD for 60 min then reperfusion for 120 min	Pentobarbital sodium (2.5%)	Gavaged with Tan IIA (30 mg/kg*d), once a day, for 7 days, before establishing model	Intravenous injected with isasteric normal saline, once a day, for 7 days, before establishing model	1. Apoptosis index	1. *p* < 0.05
							2. Caspase-3	2. *p* < 0.05
							3. Bcl-2	3. *p* < 0.01
Zhang and Zhang. 2010	Wistar rats (male/female, 20/20)	Not mentioned	Ligation of LAD for 30 min then reperfusion for 120 min	Diethyl ether	Gavaged with Tan IIA (15 mg/kg*d), once a day, for 3 days, before establishing model	Gavaged with normal saline (5 mL/kg), once a day, for 3 days, before establishing model	1. Infarct size (AAI/LVA)	1. *p* < 0.05
							2. CK	2. *p* < 0.05
							3. LDH	3. *p* < 0.05
							4. AST	4. *p* < 0.01
							5. SOD	5. *p* < 0.01
							6. MDA	6. *p* < 0.001
							7. GSH-PX	7. *p* < 0.01
Han et al., 2016	SD rats (male, 8/8)	220–270 g	Ligation of LAD for 30 min then reperfusion for 180 min	Pentobarbital sodium (3%)	Intraperitoneal injected with Tan IIA (40 mg/kg*d), once a day, for 7 days, before establishing model	Intraperitoneal injected with isasteric distilled water, once a day, for 7 days, before establishing model	1. Apoptosis index	1. *p* < 0.05
							2. P-Akt	2. *p* < 0.05
							3. Mfn2	3. *p* < 0.05
Wu and He. 2012	SD rats (male, 10/10)	280–320 g	Ligation of LAD for 30 min then reperfusion for 120 min	10% Chloral hydrate (300 mg/kg)	Gavaged with Tan IIA (50 mg/kg*d), once a day, for 7 days, before establishing model	Gavaged with normal saline (2 mL/d), once a day, for 7 days, before establishing model	1. Infarct size (AAI/LVA)	1. *p* < 0.05
							2. HR	2. *p* < 0.05
Zhou and Yang. 2012	Wistar rats (male, 16/16)	250–300 g	Ligation of LAD for 30 min then reperfusion for 60 min	3% pentobarbital sodium (0.15 mL/100 g)	Intraperitoneal injected with Tan IIA (10 mg/kg) before establishing model	Intraperitoneal injected with isasteric normal saline before establishing model	1. Infarct size (AAI weight/whole weight)	1. *p* < 0.01
							2. Arrhythmia score	2. *p* < 0.01
							3. NF-KB	3. *p* < 0.01
							4. TNF-α	4. *p* < 0.01
Han. 2011	Wistar rats (male/female, 10/10)	150–250 g	Ligation of LAD for 45 min then reperfusion for 120 min	20% Urethane (1 g/kg)	Intraperitoneal injected with Tan IIA (16 mg/kg) before establishing model	Intraperitoneal injected with normal saline (4 mL/kg) before establishing model	1. amplitude of T wave	1. *p* < 0.05
							2. incidence of 2 h arrhythmia	2. *p* < 0.05
							3. Bcl-2OD	3. *p* < 0.05
							4. BaxOD	4. *p* < 0.05
							5. Bcl-2/Bax	5. *p* < 0.05
Li et al., 2016a	SD rats (male, 13/13)	210–250 g	Ligation of LAD for 30 min then reperfusion for 120 min	Pentobarbital sodium (60 mg/kg)	Intravenous injected with Tan IIA (20 mg/kg), before establishing model	Intravenous injected with nothing, before establishing model	1. Infarct size (AAI/AAR)	1. *p* < 0.05
							2. Apoptosis index	2. *p* < 0.05
							3. CK-MB	3. *p* < 0.05
							4. LDH	4. *p* < 0.05
							5. SOD	5. *p* < 0.05
							6. MDA	6. *p* < 0.05
							7. SDH	7. *p* < 0.05
							8. COX	8. *p* < 0.05
							9. H2O2	9. *p* < 0.05
Long et al., 2015	SD rats (male, 24/24)	200–250 g	Ligation of LAD for 45 min then reperfusion for 72 h	Pentobarbital sodium (40 mg/kg)	Intravenous injected with Tan IIA (8 mg/kg), 30 min before establishing model and 24, 48 h after establishing model	Intravenous injected with isasteric normal saline, 30 min before establishing model and 24, 48 h after establishing model	1. Infarct size (AAI/AAR)	1. *p* < 0.001
							2. EF	2. *p* < 0.001
							3. FS	3. *p* < 0.001
Pan et al., 2017	SD rats (male, 9/10)	Not mentioned	Ligation of LAD for 30 min then reperfusion for 24 h	10% Chloral hydrate (350 mg/kg)	Intraperitoneal injected with Tan IIA (70 mg/kg), before establishing model	Intraperitoneal injected with isasteric normal saline, before establishing model	1. Infarct size (AAI/AAR)	1. *p* < 0.05
							2. Caspase-3	2. *p* < 0.05
							3. Bcl-2	3. *p* < 0.05
							4. Bax	4. *p* < 0.05
							5. Beclin-1	5. *p* < 0.05
							6. HMGB1	6. *p* < 0.05
Hu et al., 2015	SD rats (male, 10/10)	200–220 g	Ligation of LAD for 30 min then reperfusion for 120 min	Pentobarbital sodium (50 mg/kg)	Intraperitoneal injected with Tan IIA (40 mg/kg*d), once a day, for 14 days, before establishing model	Intraperitoneal injected with vehicle, once a day, for 14 days, before establishing model	1. Infarct size (AAI weight/whole weight)	1. *p* < 0.05
							2. CK	2. *p* < 0.05
							3. AST	3. *p* < 0.05
							4. SOD	4. *p* < 0.05
							5. GSH-Px	5. *p* < 0.05
							6. MDA	6. *p* < 0.05
							7. TNF-α	7. *p* < 0.05
							8. IL-6	8. *p* < 0.05
							9. iNOS	9. *p* < 0.05
							10. HMGB1	10. *p* < 0.05
Wei et al., 2014	SD rats (male, 8/8)	250–300 g	Ligation of LAD for 30 min then reperfusion for 24 h	Pentobarbital sodium (50 mg/kg)	Intravenous injected with Tan IIA (8 mg/kg), 4 h after reperfusion	Intravenous injected with isasteric normal saline, 4 h after reperfusion	1. Infarct size (AAI/AAR)	1. *p* < 0.05
							2. CK-MB	2. *p* < 0.05
							3. AST	3. *p* < 0.05
							4. LDH	4. *p* < 0.05
							5. SOD	5. *p* < 0.05
							6. MDA	6. *p* < 0.05
							7. GSH-Px	7. *p* < 0.05
							8. GSH	8. *p* < 0.05
Li et al., 2010	Wistar rats (male, 8/8)	250–300 g	Ligation of LAD for 30 min then reperfusion for 60 min	10% Chloral hydrate (0.4 mL/100 g)	Tail intravenous injected with Tan IIA (20 mg/kg), before establishing model	Tail intravenous injected with nothing before establishing model	1. Arrhythmia score	1. *p* < 0.01
							2. IL-6	2. *p* < 0.01
Fu et al., 2007	SD rats (male, 13/13)	280–300 g	Ligation of LAD for 45 min then reperfusion for 240 min	Diethyl ether	Gavaged with Tan IIA (60 mg/kg*d), once a day, for 14 days, before establishing model	Gavaged with 0.5% carboxymethyl cellulose sodium (10 mg/kg*d), once a day, for 14 days, before establishing model	1. SOD	1. *p* < 0.05
							2. MDA	2. *p* < 0.05
							3. Bcl-2	3. *p* < 0.05
							4. Bax	4. *p* < 0.05
							5. Caspase-3	5. *p* < 0.05
Zhang et al., 2010	SD rats (male, 20/20)	200–220 g	Ligation of LAD for 30 min then reperfusion for 180 min	3% isoflurane	Intraperitoneal injected with Tan IIA (5 mg/kg*d), once a day, for 7 days, before establishing model	Intraperitoneal injected with isasteric normal saline, once a day, for 7 days, before establishing model	1. Infarct size (AAI/LVA)	1. *p* < 0.05
							2. EF	2. *p* < 0.05
							3. TNF-α	3. *p* < 0.05
							4. IL-6	4. *p* < 0.05
							5. Caspase-3	5. *p* < 0.05
							6. P-Akt	6. *p* < 0.05
							7. NF-KB	7. *p* < 0.05
Li et al., 2016b	Wistar rats (male/female, 8/8)	130–230 g	Ligation of LAD for 30 min then reperfusion for 30 min. This process was repeated 3 times	2% Pentobarbital sodium	Tail intravenous injected with Tan IIA (2 mL/100 g*d), once a day, for 14 days, before establishing model	Tail intravenous injected with isasteric normal saline, once a day, for 14 days, before establishing model	1. SOD	1. *p* < 0.05
							2. MDA	2. *p* < 0.05
							3. CAT	3. *p* < 0.05
							4. VEGF	4. *p* < 0.05
							5. HIF-1	5. *p* < 0.05
							6. FLK-1	6. *p* < 0.05
Wang 2021	Wistar rats (male/female, 8/8)	355.62 ± 20.3 g	Ligation of LAD for 45 min then reperfusion for 24 h	3% Pentobarbital sodium	Intravenous injected with Tan IIA (20 mg/kg), before establishing model	Intravenous injected with isasteric normal saline, before establishing model	1. AAI/AAR	1. *p* < 0.05
							2. LDH	2. *p* < 0.05
							3. apoptosis index	3. *p* < 0.05
							4. ROS	4. *p* < 0.05
							5. MDA	5. *p* < 0.05
							6. SOD	6. *p* < 0.05
							7. Bcl-2	7. *p* < 0.05
Li et al., 2017	SD rats (male, 9/9)	200–300 g	Ligation of LAD for 30 min then reperfusion for 2 h	Chloral hydrate	Intraperitoneal injected with Tan IIA (2 mL/kg). The Tan IIA was administered once every other day for 8 weeks, after establishing model	Intraperitoneal injected with normal saline (0.5 mL/kg). The normal saline was administered once every other day for 8 weeks, after establishing model	1. MDA	1. *p* < 0.05
							2. SOD	2. *p* < 0.05
							3. LDH	3. *p* < 0.05
							4. Bax	4. *p* < 0.05
							5. Bcl-2	5. *p* < 0.05
							6. apoptosis index	6. *p* < 0.05
							7. CTnI	7. *p* < 0.05
Shen et al., 2018	SD rats (male, 7/7)	200–210 g	Ligation of LAD for 45 min then reperfusion for 2 h	10% chloral hydrate	Gavaged with Tan IIA (7.912 mg/kg), before establishing model	Gavaged with nothing, before establishing model	1. AAI/AAR	1. *p* < 0.05
							2. LDH	2. *p* < 0.05
							3. SOD	3. *p* < 0.05
							4. MDA	4. *p* < 0.05
Tao et al., 1996	Rabbits (male/female, 10/10)	Not mentioned	Ligation of LAD for 30 min then reperfusion for 30 min	3% pentobarbital sodium	Intravenous injected with Tan IIA (5 mg/kg), before reperfusion	Intravenous injected with nothing, before reperfusion	1. SOD	1. *p* < 0.001
							2. MDA	2. *p* < 0.001
Wang et al., 2016	New Zealand rabbits (male, 10/10)	2.2–2.7 Kg	Ligation of LMB for 90 min then reperfusion for 120 min	3% Pentobarbital sodium (1.1 mL/kg)	Intravenous injected with Tan IIA (5 mg/kg), before reperfusion	Intravenous injected with 25% Glu (2 mL/kg), before reperfusion	1. Infarct size (AAI/LVA)	1. *p* < 0.05
							2. CK	2. *p* < 0.05
							3. CK-MB	3. *p* < 0.01
							4. TNF-α	4. *p* < 0.05
Ai 2013	Rabbits (male/female, 10/10)	2.0–2.5 Kg	Ligation of LCA for 30 min then reperfusion for 120 min	20% Urethane (5 mL/kg)	Intravenous injected with Tan IIA (3 mg/kg), before establishing model	Intravenous injected with nothing before establishing model	1. SOD	1. *p* < 0.05
							2. MDA	2. *p* < 0.05
Wang et al., 2012	New Zealand rabbits (male, 10/10)	2.2–2.7 Kg	Ligation of LMB for 90 min then reperfusion for 120 min	3% Pentobarbital sodium (1.1 mL/kg)	Intravenous injected with Tan IIA (5 mg/kg), before reperfusion	Intravenous injected with 25% Glu (2.0 mL/kg), before reperfusion	1. CTnI	1. *p* < 0.01
							2. SOD	2. *p* < 0.05
Lin et al., 2013	New Zealand rabbits (male, 10/10)	2.5–3.0 Kg	Ligation of LVA for 60 min then reperfusion for 120 min	3% Pentobarbital sodium (1.0 mL/kg)	Intravenous injected with Tan IIA (4 mg/kg), before reperfusion	Intravenous injected with isasteric normal saline, before reperfusion	1. Infarct size (AAI/LVA)	1. *p* < 0.05
							2. ST -segment changes	2. *p* < 0.05
Wang et al., 2007	Japan-Sino hybridization whit e rabbits (male/female, 8/6)	2.28 ± 0.31 Kg	Ligation of LCA for 40 min then reperfusion for 60 min	Pentobarbital sodium (30 mg/kg)	Intravenous injected with Tan IIA (3 mg/kg), before establishing model	Intravenous injected with 25% Glu (1.0 mL/kg), before establishing model	1. CK	1. *p* < 0.01
							2. LDH	2. *p* < 0.01
							3. SOD	3. *p* < 0.05
							4. GSH-Px	4. *p* < 0.05
							5. MDA	5. *p* < 0.05
Liu et al., 2017	New Zealand rabbits (male/female, 10/10)	1.5–1.8 Kg	Ligation of LCA for 30 min then reperfusion for 30 min. This process was repeated 3 times	Pentobarbital sodium (1 mL/kg)	Intravenous injected with Tan IIA (5 mL/kg*d), once a day, for 7 days, before establishing model	Intravenous injected with isasteric normal saline, once a day, for 7 days, before establishing model	1. CK-MB	1. *p* < 0.05
							2. LDH	2. *p* < 0.05
							3. SOD	3. *p* < 0.05

Note: AAI, area at infarct; AAR, area at risk; AST, aspartate transaminase; Bcl-2, B-cell lymphoma-2; Bax, Bcl-2-Associated X; cTnI, cardiac troponin I; CytC, cytochrome C; CAT, catalase; CK, creatine kinase; CK-MB, creatine kinase-MB; COX, cytochrome c oxidase; EF, ejection fraction; FS, fractional shorting; Glu, glucose injection; GSH, glutathione synthetase; GSH-Px, glutathione peroxidase; HMGB1, high mobility group box-1 protein; HO-1, hemeoxygenase-1; iNOS, inducible nitric oxide synthase; IL-6, interleukin-6; LAD, the left anterior descending coronary artery; LMB, the left marginal branch; LCA, the left circumflex coronary artery; LVA, the left ventricular coronary artery; LVA, left ventricular area; LDH, lactate dehydrogenase; MDA, malondialdehyde; Nrf-2, nuclearfactor erythroid-2-related factor-2; Mfn2, mitofusin2; p-Akt, phosphothreonine kinase; SD rats, Sprague-Dawley; SOD, superoxide dismutase; TNF-α, tumor necrosis factor-α.

### 3.2 Study quality

The quality score of the studies ranged from 3 to 7. All studies were published in a peer-reviewed journal. They all described random allocation to treatment or control and used an anesthetic without substantial intrinsic vascular protection activity. Eleven studies (Fu et al., 2007; Fu et al., 2007; Zhang et al., 2010; Wei et al., 2014; Hu et al., 2015; Long et al., 2015; Li Q. et al., 2016; Li X. Y. et al., 2016; Liu et al., 2017; Pan et al., 2017; Tang et al., 2017) reported control of temperature. One study (Zhang et al., 2010) mentioned an appropriate animal model. Eleven studies (Fu et al., 2007; Fu et al., 2007; Zhang et al., 2010; Wei et al., 2014; Hu et al., 2015; Long et al., 2015; Li Q. et al., 2016; Li X. Y. et al., 2016; Liu et al., 2017; Pan et al., 2017; Tang et al., 2017) stated compliance with animal welfare regulations, and 10 studies (Fu et al., 2007; Fu et al., 2007; Zhang et al., 2010; Hu et al., 2015; Long et al., 2015; Li Q. et al., 2016; Li X. Y. et al., 2016; Pan et al., 2017; Li et al., 2019) a statement of potential conflicts of interest. None of the studies mentioned blinded induction of the model, blinded assessment of outcome or sample size calculation. The methodological quality is shown in Table 2.

**TABLE 2:** Risk of bias of the included studies.

Study	A	B	C	D	E	F	G	H	I	J	Total
Ma et al., 2017	**√**		**√**			**√**					3
Dai et al., 2013	**√**		**√**			**√**					3
Tang et al., 2017	**√**	**√**	**√**			**√**			**√**	**√**	6
Zheng et al., 2010	**√**		**√**			**√**					3
Zhang and Zhang. 2010	**√**		**√**			**√**					3
Han et al., 2016	**√**		**√**			**√**				**√**	4
Wu and He. 2012	**√**		**√**			**√**				**√**	4
Zhou and Yang. 2012	**√**		**√**			**√**				**√**	4
Han. 2011	**√**		**√**			**√**					3
Li et al., 2016a	**√**	**√**	**√**			**√**			**√**	**√**	6
Long et al., 2015	**√**	**√**	**√**			**√**			**√**	**√**	6
Pan et al., 2017	**√**	**√**	**√**			**√**			**√**	**√**	6
Hu et al., 2015	**√**	**√**	**√**			**√**			**√**	**√**	6
Wei et al., 2014	**√**	**√**	**√**			**√**			**√**		5
Li et al., 2010	**√**		**√**			**√**				**√**	4
Fu et al., 2007	**√**	**√**	**√**			**√**			**√**	**√**	6
Zhang et al., 2010	**√**	**√**	**√**			**√**	**√**		**√**	**√**	7
Li et al., 2016b	**√**	**√**	**√**			**√**			**√**	**√**	6
Wang 2021	**√**		**√**			**√**					3
Li et al., 2017	**√**		**√**			**√**				**√**	4
Shen et al., 2018	**√**	**√**	**√**			**√**			**√**	**√**	6
Tao et al., 1996	√		√			√					3
Wang et al., 2016	√		√			√				**√**	4
Ai 2013	√		√			√					3
Wang et al., 2012	√		√			√				**√**	4
Lin et al., 2013	√		√			√				**√**	4
Wang et al., 2007	√		√			√					3
Liu et al., 2017	**√**	**√**	**√**			√			**√**	**√**	6

Note: Studies fulfilling the criteria of: A: peer reviewed publication; B: control of temperature; C: random allocation to treatment or control; D: blinded induction of model; E: blinded assessment of outcome; F: use of anesthetic without significant intrinsic cardioprotective activity; G: appropriate animal model (aged, diabetic, or hypertensive); H: sample size calculation; I: compliance with animal welfare regulations; J: statement of potential conflict of interests. Compliant item was given one point using a tick to indicate.

### 3.3 Effectiveness

#### 3.3.1 Primary outcome measures

##### 3.3.1.1 Myocardial infarct size

**FIGURE 3:**
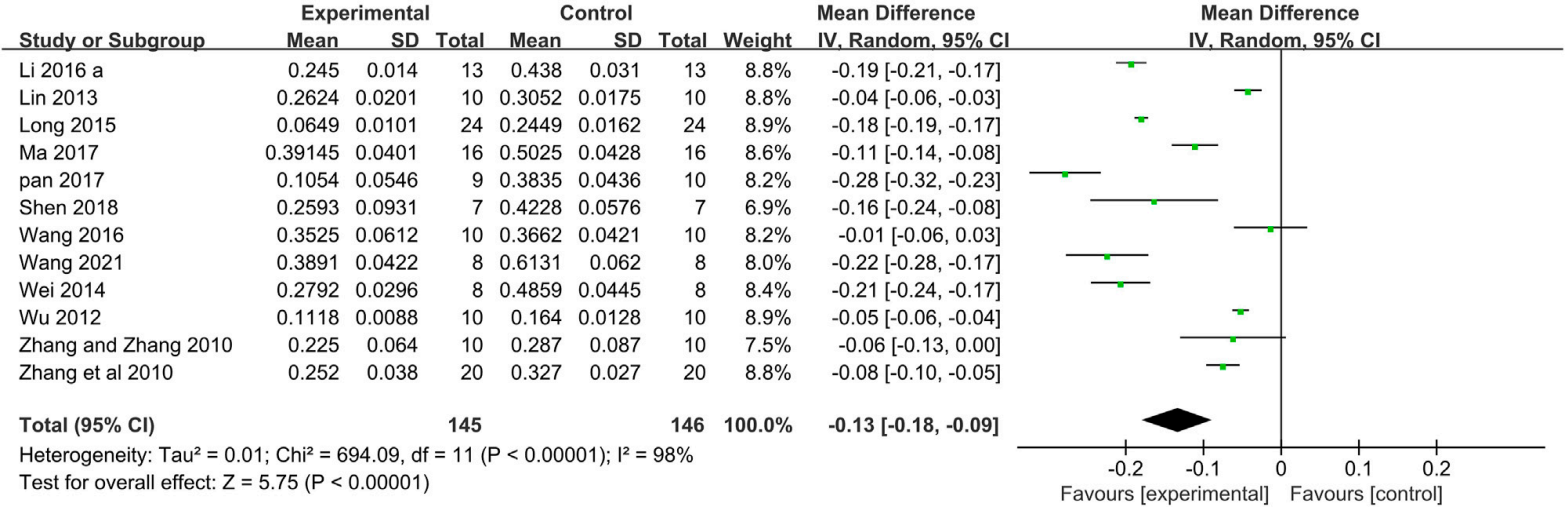
The forest plot: effects of Tan IIA for decreasing the myocardial infarction size compared with control group.

**FIGURE 4:**
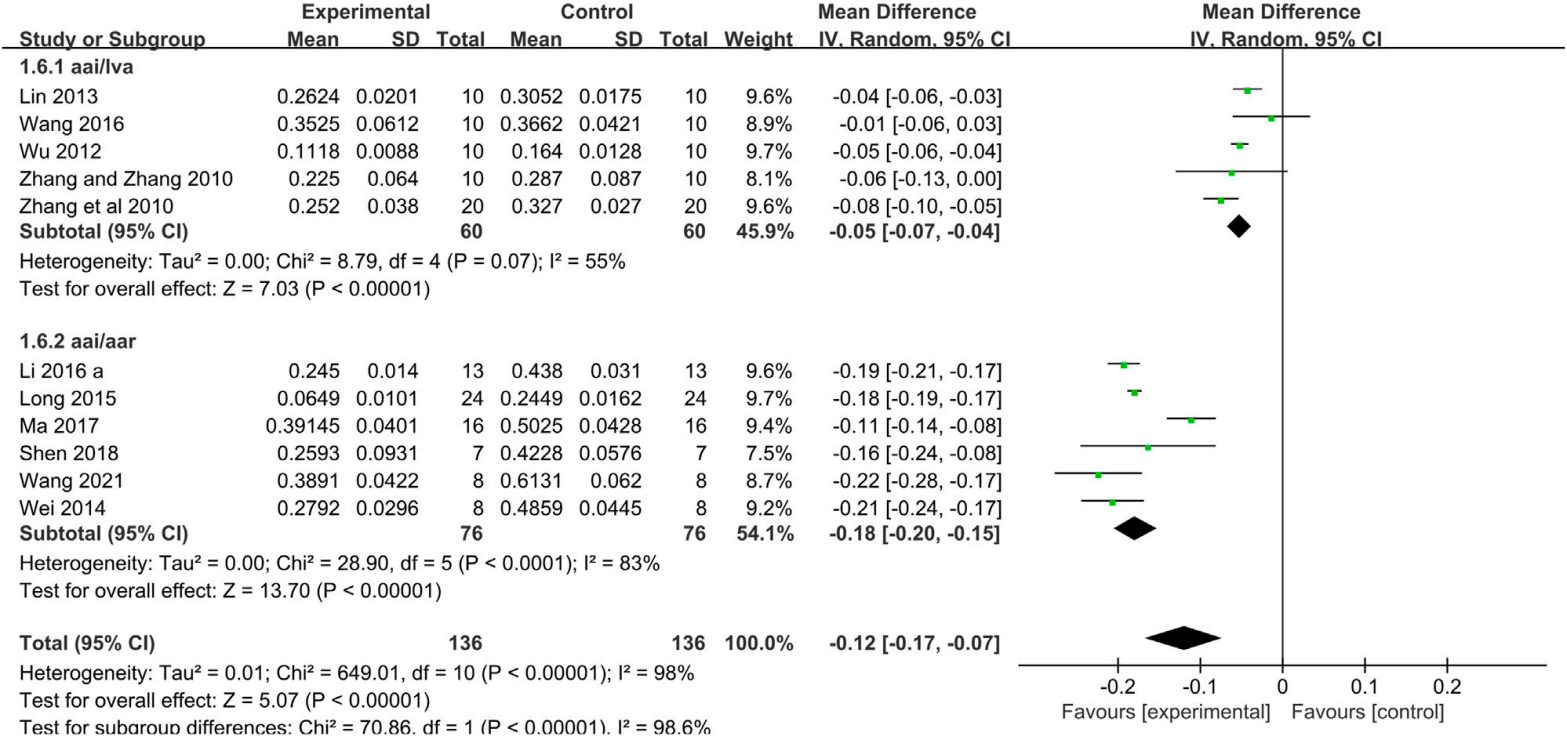
The forest plot: effects of Tan IIA for reducing the myocardial infarction size (area at infarct/left ventricular area, area at infarct/area at risk) compared with control group.

**FIGURE 5:**
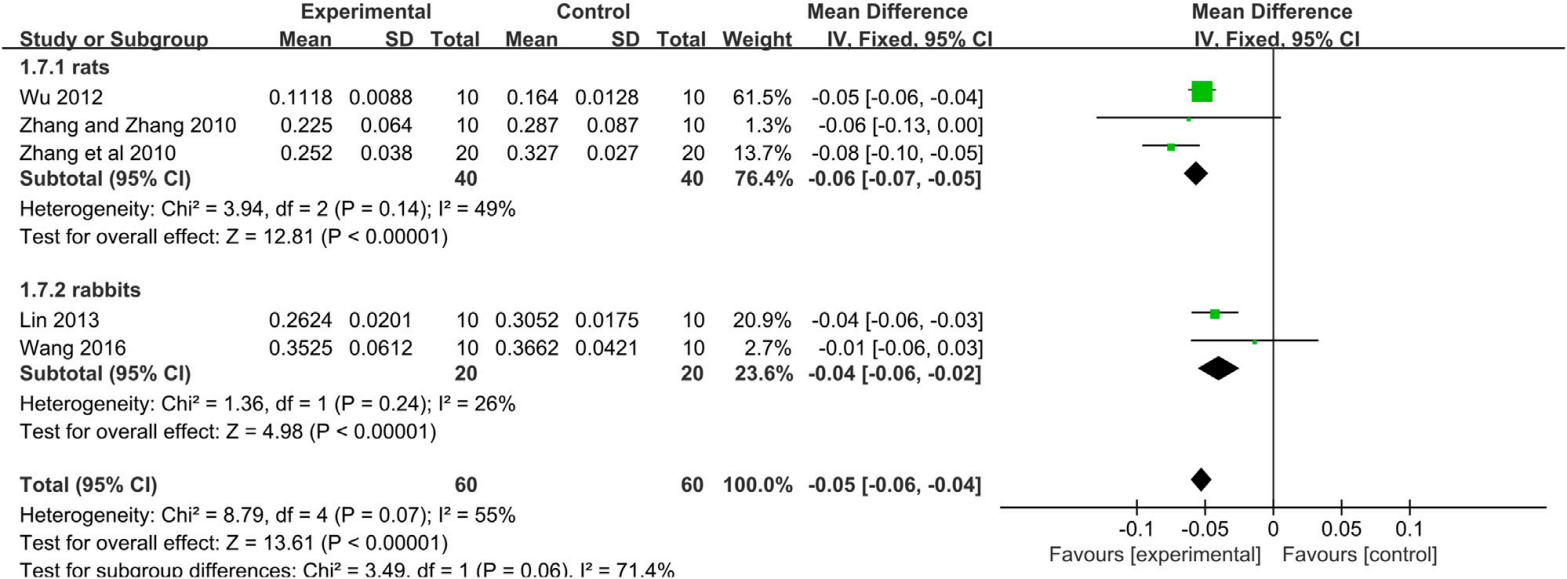
The forest plot: effects of Tan IIA for reducing the myocardial infarction size in rats/rabbits compared with control group.

**FIGURE 6:**
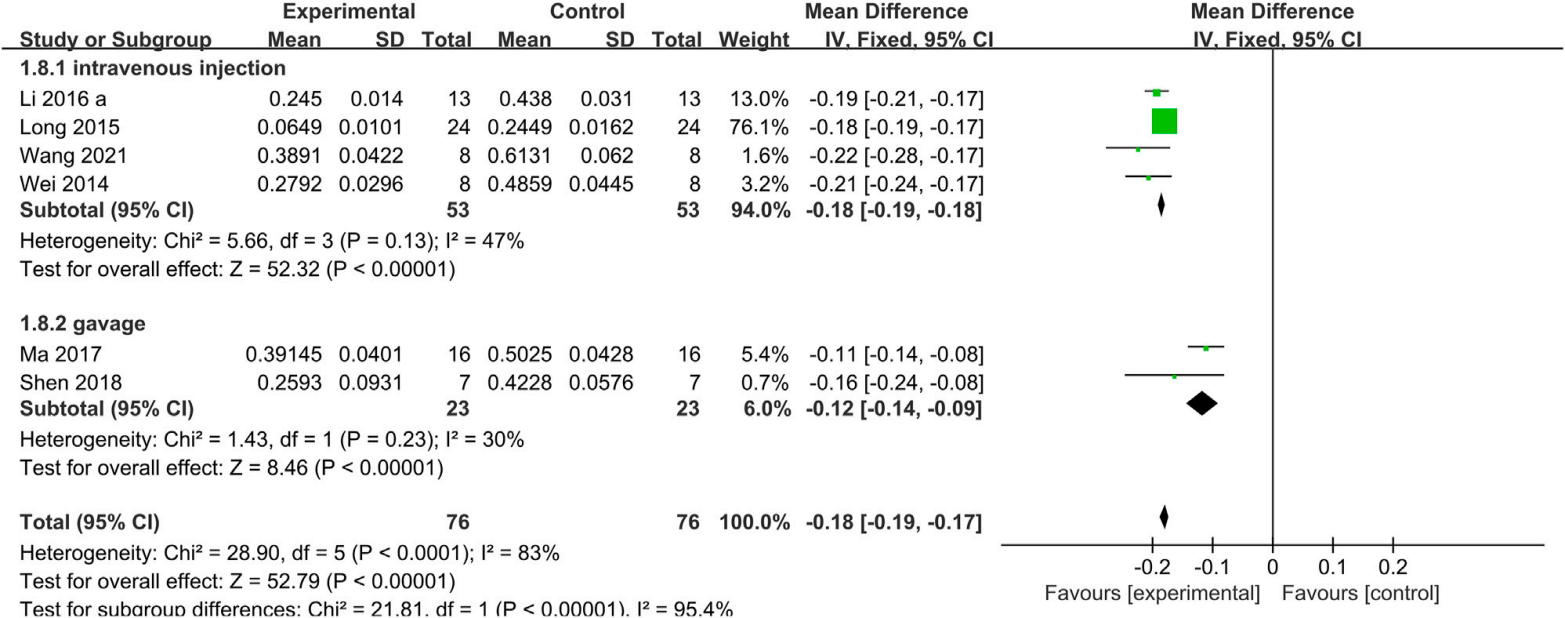
The forest plot: effects of Tan IIA for reducing the myocardial infarction size in different routes of administration group compared with control.

Twelve studies (Zhang et al., 2010; Zhang and Zhang, 2010; Wu and He, 2012; Lin et al., 2013; Wei et al., 2014; Long et al., 2015; Li Q. et al., 2016; Wang et al., 2016; Ma et al., 2017; Pan et al., 2017; Shen et al., 2018; Wang, 2021) used myocardial infarct size as an outcome measure. Meta-analysis of 12 studies that the Tan IIA-treated group exhibited significantly decreased myocardial infarct size compared with that of the control group (*n* = 145, MD −0.13, 95% CI [−0.18 to −0.09], *p < 0.00001*; *I* ^*2*^ *= 98%*) (Figure 3). Owing to obvious heterogeneity, we conducted subgroup analysis according to the different calculation methods. 1) The meta-analysis of 5 studies (Zhang et al., 2010; Zhang and Zhang, 2010; Wu and He, 2012; Lin et al., 2013; Wang et al., 2016) showed that the Tan IIA-treated group exhibited a significantly decreased the area at infarct/left ventricular area (AAI/LVA) compared with that of the control group (*n* = 60, MD −0.05, 95% CI [−0.07 to −0.04], *p* = *0.07*; *I* ^*2*^ *= 55%*) (Figure 4); The meta-analysis of 7 studies (Fu et al., 2007; Wei et al., 2014; Long et al., 2015; Li Q. et al., 2016; Ma et al., 2017; Shen et al., 2018; Wang, 2021) that Tan IIA significantly reduced the area at infarct/area at risk (AAI/AAR) (*n* = 76, MD −0.18, 95% CI [−0.20 to −0.15], *p < 0.0001*; *I* ^*2*^ *= 83%*) (Figure 4). Although the heterogeneity decreased after subgroup analysis, the heterogeneity in both subgroups was still high. Thus, further subgroup analysis was performed in the AAI/LVA and AAI/AAR groups. In the AAI/LVA studies, the different animal species (rats or rabbits) may have been the source of heterogeneity. The meta-analysis of 3 rats studies (Zhang et al., 2010; Zhang and Zhang, 2010; Wu and He, 2012) showed a significant effect of Tan IIA on decreasing the AAI/LVA (*n* = 40, MD −0.06, 95% CI [−0.07 to −0.05], *p* = *0.14*; *I* ^*2*^ *= 49%*) (Figure 5), and the meta-analysis of 2 rabbit studies (Lin et al., 2013; Wang et al., 2016) showed similar results (*n* = 20, MD −0.04, 95% CI [−0.06 to −0.02], *p* = *0.24*; *I* ^*2*^ *= 26%*) (Figure 5). In the AAI/AAR studies, different routes of administration (intravenous injection and gavage) may have been the source of heterogeneity. The meta-analysis of the 4 studies (Wei et al., 2014; Long et al., 2015; Li Q. et al., 2016; Wang, 2021) that used intravenous injection indicated a significant effect of Tan IIA on reducing the AAI/AAR (*n* = 53, MD −0.18, 95% CI [−0.19 to −0.18], *p* = *0.13*; *I* ^*2*^ *= 47%*) (Figure 6), and the meta-analysis on the 2 studies (Ma et al., 2017; Shen et al., 2018) that used gavage administration showed similar results (*n* = 23, MD −0.12, 95% CI [−0.14 to −0.09], *p* = *0.23*; *I* ^*2*^ *= 30%*) (Figure 6).

##### 3.3.1.2 Cardiac enzymes and troponin

**FIGURE 7:**
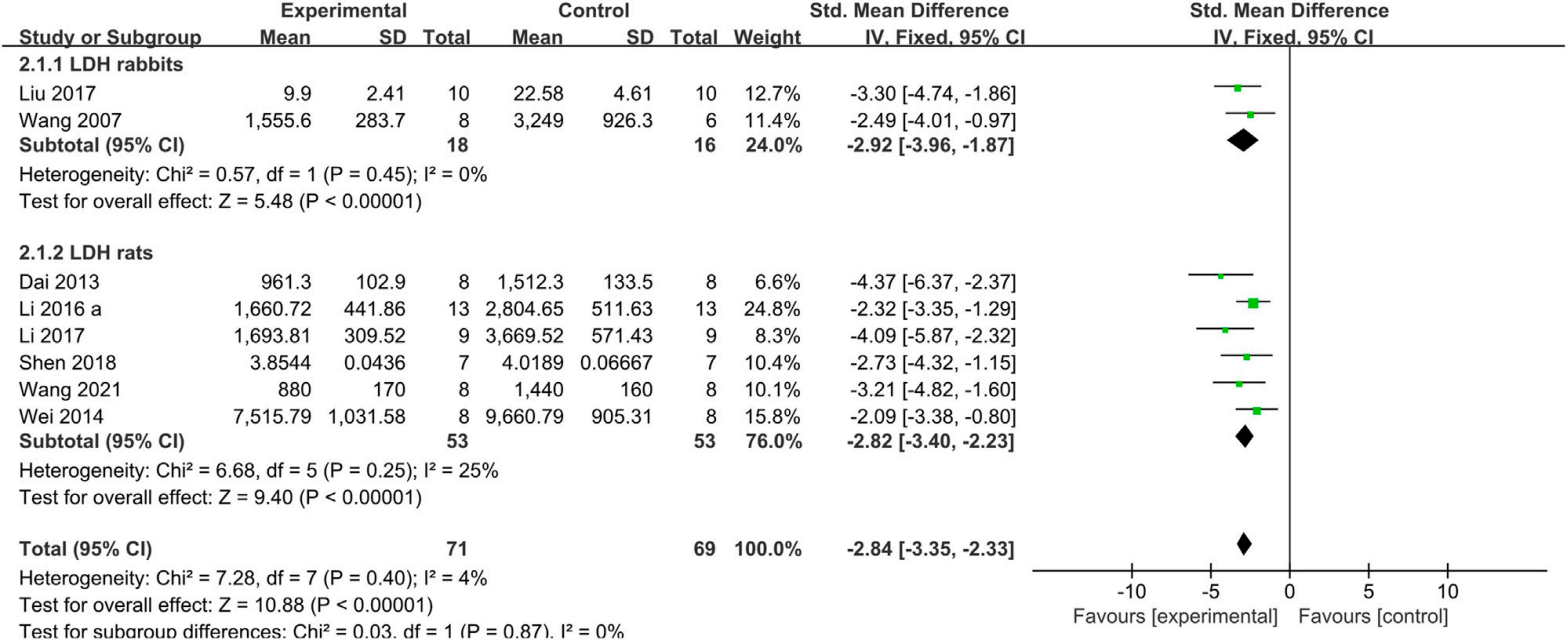
The forest plot: effects of Tan IIA for decreasing lactate dehydrogenase in rats/rabbits compared with control group.

**FIGURE 8:**
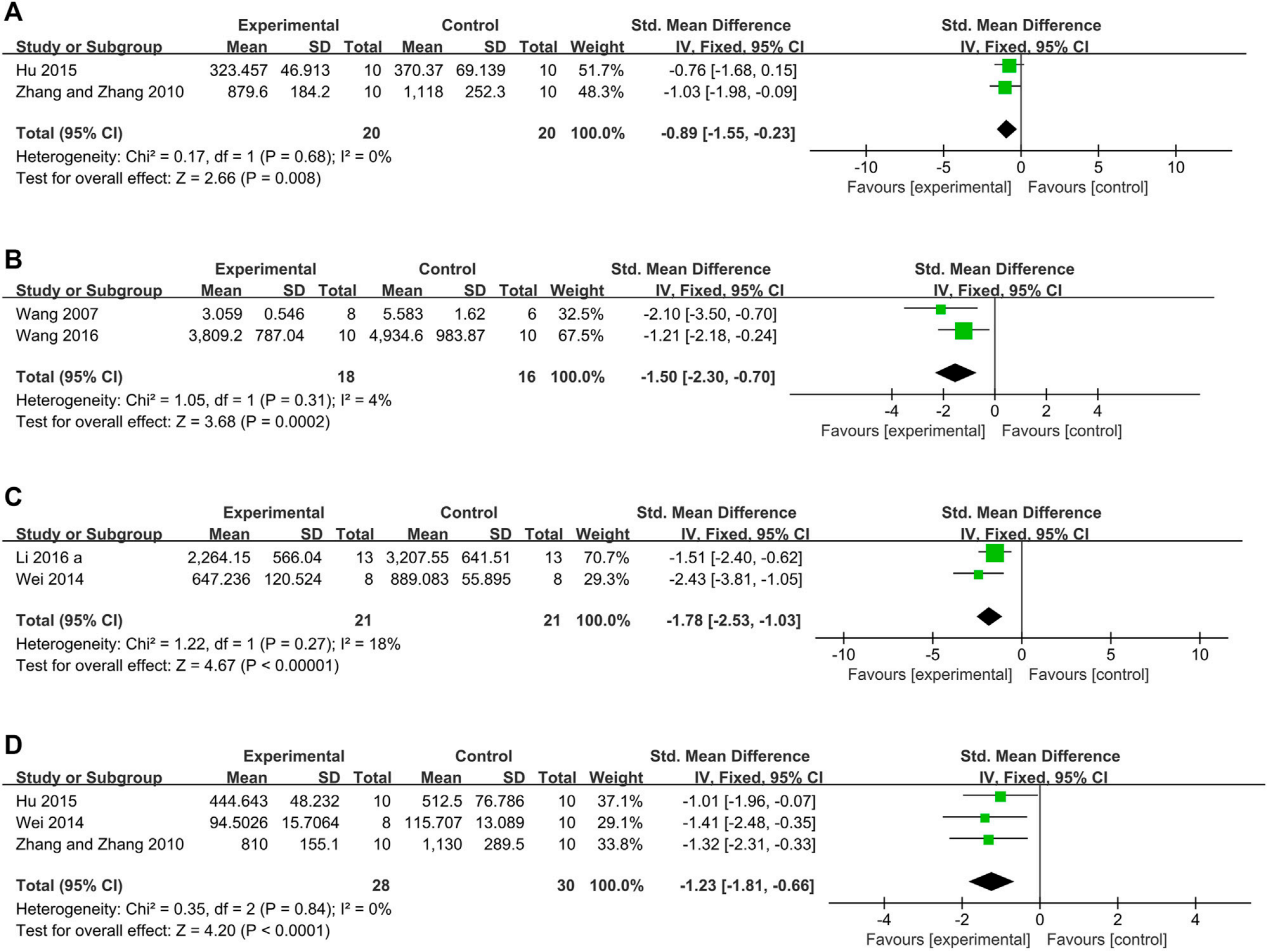
The forest plot: effects of Tan IIA for decreasing creatine kinase **(A)** rats group; **(B)** rabbits group; **(C)** creatine kinase-MB; **(D)** aspartate transaminase compared with control group.

The meta-analysis of 9 studies (Fu et al., 2007; Wang et al., 2007; Zhang and Zhang, 2010; Dai et al., 2013; Wei et al., 2014; Li Q. et al., 2016; Liu et al., 2017; Li et al., 2019; Wang, 2021) showed that the Tan IIA-treated group exhibited a significant decrease in LDH levels compared with those in the control group (*n* = 81, SMD −2.49, 95% CI [−2.94 to −2.04], *p* = *0.05*; *I* ^*2*^ *= 49%*). As a result of the obvious heterogeneity, we conducted subgroup analysis according to the different animal species (rats or rabbits). The two rabbit studies (Wang et al., 2007; Liu et al., 2017) showed a significant effect of Tan IIA on decreasing LDH (*n* = 16, SMD −2.92, 95% CI [−3.96 to −1.87], *p = 0.45*; *I* ^*2*^ *= 0%*) (Figure 7). The seven rat studies (Fu et al., 2007; Zhang and Zhang, 2010; Dai et al., 2013; Wei et al., 2014; Li Q. et al., 2016; Li et al., 2019; Wang, 2021) showed a significant effect of Tan IIA on decreasing LDH (*n* = 63, SMD −2.39, 95% CI [−2.89 to −1.89], *p* = *0.03*; *I* ^*2*^ *= 58%*). We performed a sensitivity analysis by sequentially excluding each study in the rat group. After removing one study (Zhang and Zhang, 2010) in which the dosage of Tan IIA administered before establishing the model was much higher than that in other studies, the meta-analysis of the remaining 6 rat studies (Fu et al., 2007; Dai et al., 2013; Wei et al., 2014; Li Q. et al., 2016; Li et al., 2019; Wang, 2021) showed that Tan IIA decreased LDH levels (*n* = 53, SMD −2.82, 95% CI [−3.40 to −2.23], *p* = *0.25*; *I* ^*2*^ *= 25%*) (Figure 7). Three rat studies (Zhang and Zhang, 2010; Dai et al., 2013; Hu et al., 2015) used CK as the outcome metric. After removing the study (Dai et al., 2013) in which the ligation time was distinct from that in the other studies, the meta-analysis of the 2 remaining studies (Zhang and Zhang, 2010; Hu et al., 2015) showed that the Tan IIA-treated group exhibited significantly decreased CK levels compared with those in the control group (*n* = 20, SMD −0.89, 95% CI [−1.55 to−0.23], *p* = *0.68*; *I* ^*2*^ *= 0%*) (Figure 8A). Moreover, the meta-analysis of 2 rabbit studies (Wang et al., 2007; Wang et al., 2016) showed that Tan IIA reduced CK levels (*n* = 18, SMD −1.51, 95% CI [−2.33 to −0.68], *p* = *0.31*; *I* ^*2*^ *= 4%*) (Figure 8B). Two studies (Wei et al., 2014; Li Q. et al., 2016) showed that the Tan IIA-treated group had decrease CK-MB levels compared with those in the control group (*n* = 21, SMD −1.78, 95% CI [−2.53 to −1.03], *p* = *0.27*; *I* ^*2*^ *= 18%*) (Figure 8C). The meta-analysis of 3 studies showed (Zhang and Zhang, 2010; Wei et al., 2014; Hu et al., 2015) that the Tan-IIA treated group exhibited significantly reduced AST levels compared with those in the control group (*n* = 28, SMD −1.23, 95% CI [−1.81 to −0.66], *p* = *0.84*; *I* ^*2*^ *= 0%*) (Figure 8D). One study (Ma et al., 2017) used cTnI as the outcome measure and showed that the Tan IIA-treated group exhibited decreased cTnI levels compared with those in the control group (*p < 0.05*).

##### 3.3.1.3 The level of ST-segment elevation and cardiac function

One study (Li et al., 2019) reported that compared to the control, Tan IIA can reduce ST-segment elevation (*p < 0.05*) and 2 studies (Zhang et al., 2010; Long et al., 2015) showed that Tan IIA-treated group had significantly improved left ventricular ejection fraction (LVEF) compared with that in the control group (*p < 0.05*).

#### 3.3.2 Secondary outcome measures

**FIGURE 9:**
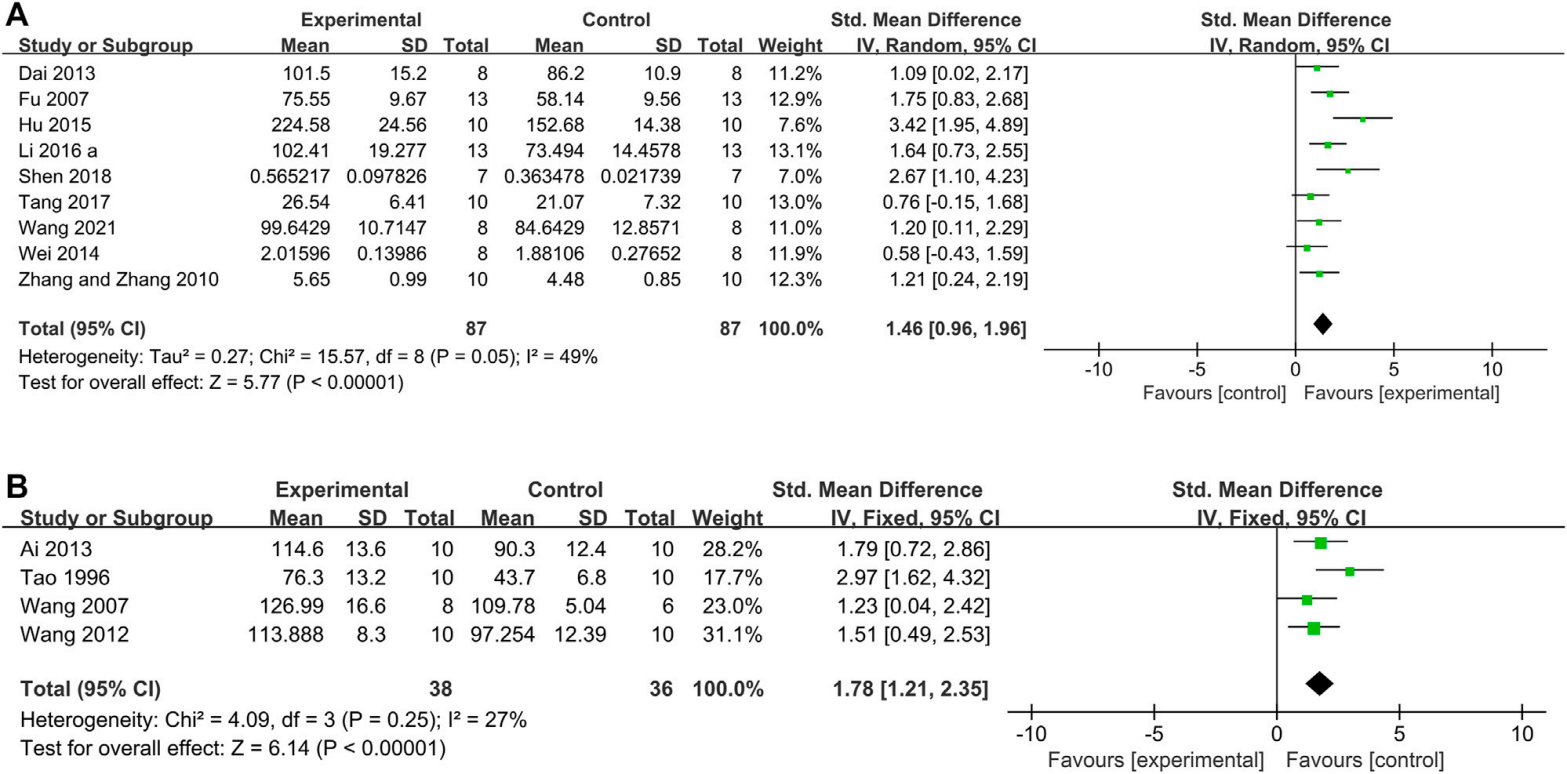
The forest plot: effects of Tan IIA for increasing superoxide dismutase compared with control group. **(A)** Rats group; **(B)** rabbits group.

**FIGURE 10:**
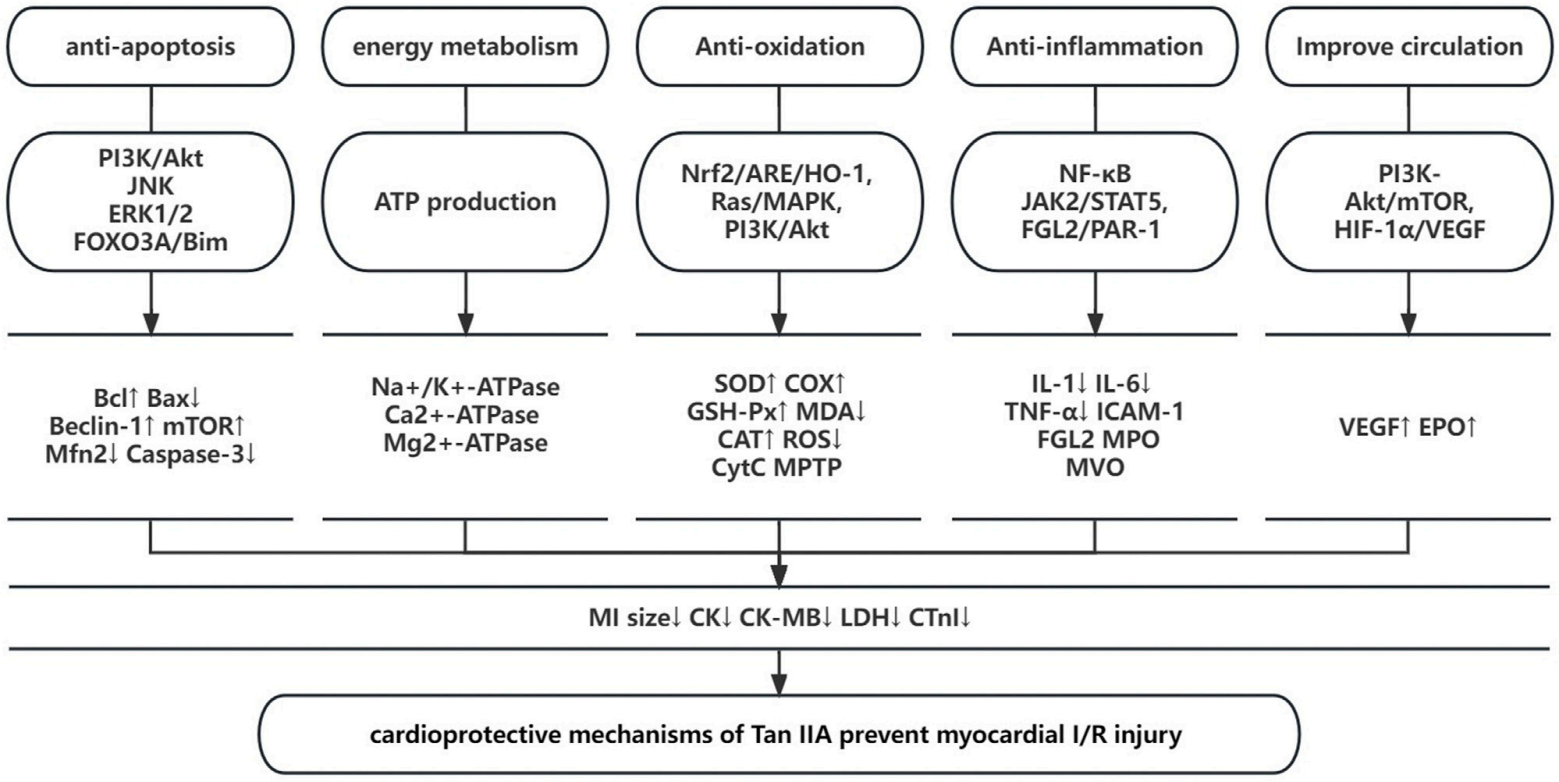
A schematic representation of cardioprotective mechanisms of Tan IIA for myocardial ischemia/reperfusion injury.

The meta-analysis of 9 studies (Fu et al., 2007; Fu et al., 2007; Zhang and Zhang, 2010; Dai et al., 2013; Wei et al., 2014; Hu et al., 2015; Li Q. et al., 2016; Tang et al., 2017; Wang, 2021) showed that the Tan IIA-treated group rats exhibited significantly increased SOD levels compared to those in control group rats (*n* = 87, SMD 1.46, 95% CI [0.96 to 1.96]; *p* = *0.05*; *I* ^*2*^ *= 49%*) (Figure 9A); 4 studies (Wang et al., 2007; Tan et al., 1996; Wang et al., 2012; Ai, 2013; Wang et al., 2016; Li et al., 2019) showed that Tan IIA increased the SOD levels in rabbits (*n* = 38, SMD 1.80, 95% CI [1.14 to 2.47], *p* = *0.25*; *I* ^*2*^ *= 27%*) (Figure 9B); 13 studies (Tao et al., 1996; Fu et al., 2007; Wang et al., 2007; Zhang and Zhang, 2010; Ai, 2013; Dai et al., 2013; Wei et al., 2014; Hu et al., 2015; Li Q. et al., 2016; Liu et al., 2017; Tang et al., 2017; Shen et al., 2018; Wang, 2021) showed that Tan IIA reduced MDA levels (*n* = 137, SMD −3.40, 95% CI [−4.39 to −2.41]; *p < 0.00001*; *I* ^*2*^ *= 85%*); 5 studies (Zhang and Zhang, 2010; Wang et al., 2012; Wei et al., 2014; Hu et al., 2015; Tang et al., 2017) showed that Tan IIA increased GSH-Px levels; 5 studies (Zheng et al., 2010; Dai et al., 2013; Li Q. et al., 2016; Han et al., 2016; Wang, 2021) that Tan IIA decreased the myocardial cell apoptotic index; 4 studies (Fu et al., 2007; Zhang et al., 2010; Zheng et al., 2010; Pan et al., 2017) showed that Tan IIA reduced caspase-3 levels (*p < 0.05*); 1 study (Li Q. et al., 2016) showed that Tan IIA increased the expression levels of PI3K (*p* < 0.05) and p-Akt/Akt ratio; 1 study (Han et al., 2016) showed that Tan IIA increased the expression levels of p-Akt (*p < 0.05*); 4 studies (Zhang et al., 2010; Zhou and Yang, 2012; Hu et al., 2015; Wang et al., 2016) showed that Tan IIA reduced TNF-α levels (*p < 0.05*); 2 studies (Hu et al., 2015; Pan et al., 2017) showed that Tan IIA reduced HMGB1 levels; 3 studies showed that Tan IIA increased the ratio of Bcl-2/Bax proteins (Fu et al., 2007; Han, 2011; Pan et al., 2017). A schematic of the studied mechanisms by which Tan IIA protects against myocardial I/R injury is provided (Figure 10).

## 4 Discussion

### 4.1 Summary of the evidence

The present study demonstrated that Tan IIA exerted potential cardioprotective functions mainly through antioxidant, anti-inflammatory, and anti-apoptosis mechanisms and improved circulation and energy metabolism in myocardial I/R models.

### 4.2 Limitations

There are several limitations that should be considered. First, a certain degree of selective bias is inevitable because the search strategy was limited to Chinese and English databases (Nolting et al., 2012), and negative findings are rarely published (Franco et al., 2014). Second, no study mentioned calculation of sample size, blinding of model establishment and outcome measurement. Moderate methodological quality will affect the accuracy of the results (Landis et al., 2012). Third, MI generally occurs in patients with comorbidities such as old age, diabetes or hypertension (Blankstein et al., 2012), and only 1 study (Zhang et al., 2010) constructed an animal model in diabetic rats. Finally, some studies used only female animals. The sensitivity and responses of animals of different sexes to the same drug or stimuli are quite different, especially in the cardiovascular system, which could interfere with the experimental results (Barp et al., 2002; Bae and Zhang, 2005).

### 4.3 Implications

Systematic reviews of animal studies can contribute to the improvement in the methodological quality of experiments (de Vries et al., 2014). In the present study, the methodological quality was moderate. Most of the deducted points were because of failures in sample size calculation and blinding in group allocation and outcome assessment, which are considered the core standards of research design. Studies with a lower quality tended to show better outcomes, resulting in global overestimation of the effect (García-Bonilla et al., 2012). For animal research, the “Reporting *in vivo* experiments (ARRIVE) guidelines” (Kilkenny et al., 2012) were proposed and are continuously improved to provide guidance on the complete and transparent reporting of *in vivo* animal research and to improve the quality of research reports. Thus, it is suggested that higher quality study design and reporting for Tan IIA should be executed according to the ARRIVE guidelines. Moreover, some aspects, such as calculation of sample size and blinding of group allocation and outcome assessment, should be specifically focused on.

In the present study, the vast majority of the included studies used young and healthy animals. However, in contrast to these young and healthy animal models, patients with MI usually have multiple cardiovascular risk factors and comorbidities, such as hypertension, hyperlipidemia, diabetes, hyperglycemia, heart failure, altered coronary circulation, and aging (Ferdinandy et al., 2014). These cardiovascular risks and comorbidities need to be treated in both the short and long term and contribute to the development of IR injury and complicate therapy (Rossello and Yellon, 2016). In addition, inappropriate selection of animal models could contribute to spurious or inconsistent results as well as unnecessary animal use (Lecour et al., 2014). Therefore, we propose the following suggestions: 1) choosing animal models that have similar anatomy and physiology to those of humans; 2) experiments with animal models that include risk factors and comorbidities; and 3) setting endpoints of animal studies closely resembling clinical settings. Furthermore, inconsistencies were particularly obvious in congenital heart disease (CHD) studies that used animals of different sexes (Barrett-Connor, 2013). Estrogen has been observed to have a cardioprotective effect both in clinical and preclinical studies, although the specific mechanism remains to be explored (Menazza et al., 2017). We suggest that sex differences in animals should be considered in experimental design.

Infarct size is the most robust primary outcome that is invariably used in animal experimental studies to evaluate the efficacy of various pharmacological or non-pharmacological strategies in preventing reperfusion injury, and it is usually analyzed after a short-term reperfusion or after a few days in an acute experiment (Ndrepepa et al., 2017). However, it is rarely tested clinically due to the limited diagnostic means, which is not helpful for intuitively evaluating the effect of different treatment strategies on cardiac function and determining the prognosis of patients. With the development of single-photon emission computed tomography (SPECT) and cardiac magnetic resonance (CMR), we propose that patients with acute coronary syndrome, especially ST-segment elevation myocardial infarction (STEMI), should be have their infarct size measured (Gibbons and Araoz, 2016) to stratify the risk of heart failure in patients and develop appropriate treatment strategies.

Recorded in the famous Traditional Chinese Medicine Classic “Compendium of Materia Medica,” the function of *S. miltiorrhiza* is described as invigorating the circulation of blood, dredging the collateral vessels on the pericardium and heart surface and treating colic. As the major active lipophilic ingredient of *S. miltiorrhiza*, Tan IIA is considered as the main contributor to the above efficacy. In Chinese medicine theory, it is used as a drug for promoting blood circulation and resolving blood stasis for cardiovascular ischemic diseases. However, the molecular and biological mechanisms of the cardioprorective effects of Tan IIA have not been fully elucidated. Experimental research has shown that myocardial I/R injury is related to several pathophysiological features, including the inflammatory response, endothelial dysfunction, generation of oxygen free radicals, mitochondrial dysfunction, myocardial cell apoptosis and autophagy (Heusch and Gersh, 2017). According to the present study, the possible mechanisms through which Tan IIA prevents myocardial I/R injury are as follows: 1) Anti-apoptosis: Tan IIA can increase the ratio of Bcl-2/Bax, downregulate the protein expression of Bax, decrease the expression level of caspase-3, upregulate autophagic markers (Beclin-1 and the ratio of LC3B/LC3A) and downregulate the expression of Mfn2, leading to anti-apoptosis effects against myocardial I/R injury by regulating the PI3K/AKT/mTOR, Ras-PI3K/Akt, JNK, ERK1/2, and PI3K/Akt/FOXO3A/Bim pathways. 2) Improving energy metabolism: Tan IIA can increase glucose oxidation; augment ATP restoration and production; improve the activities of Na+/K^+^-ATPase, Ca^**2+**^-ATPase and Mg^**2+**^-ATPase; alleviate the consumption of energy charge; reverse Ca^**2+**^ overload; and protect the function of mitochondria. 3) Antioxidation: Tan IIA can promote the process of oxidative phosphorylation; increase the levels of antioxidants such as SOD, SDH, COX, GSH-Px, and CAT; and increase the release of NO, which simultaneously reduces free radical generation such as reactive oxidative species (O2-, HO-, and H_2_O_2_); Tan IIA also regulates the mitochondrial permeability transition pore (MTPT), suppresses chondriokinesis, and reduces the release of cytochrome c and malondialdehyde (MDA), resulting in antioxidation effects against myocardial I/R injury by regulating the Nrf2/ARE/HO-1, Ras/MAPK, and PI3K/Akt signaling pathways. 4) Anti-inflammation: Tan IIA can inhibit the expression of inflammation-related cytokines, such as IL-1, IL-6, TNF-α, and ICAM-1; upregulate the expression of EPOR; decrease the activation of NF-κB (p50 and p65); inhibit FGL2 and MPO expression; ameliorate microvascular obstruction (MVO); downregulate the mRNA expression of iNOS; and downregulate HMGB1 expression, leading to an anti-inflammatory effect against myocardial I/R injury by activating NF-κB and regulating the JAK2/STAT5 and FGL2-thrombin/PAR-1 pathways. 5) Improve circulation: Tan IIA can promote the expression of VEGF and EPO and facilitate the proliferation and differentiation of vascular cells to help vascular repair by the PI3K-Akt/mTOR and HIF-1α/VEGF pathways.

### 4.5 Conclusion

Tan IIA exerts cardioprotective function in myocardial I/R injury mainly through antioxidant, anti-inflammatory, and anti-apoptosis mechanisms and improving the circulation and energy metabolism. Thus, Tan IIA is a promising cardioprotective agent that should be further tested in MI clinical trials.

## Data Availability

The original contributions presented in the study are included in the article/supplementary material, further inquiries can be directed to the corresponding author.
